# Thoracoabdominal impalement with criminal intent

**DOI:** 10.1590/0100-3984.2016.0205

**Published:** 2018

**Authors:** Antonio Gabriel de Jesus Barbosa, Gláucia Zanetti, Edson Marchiori

**Affiliations:** 1 Universidade Federal do Rio de Janeiro (UFRJ), Rio de Janeiro, RJ, Brazil

**Figure 1:**
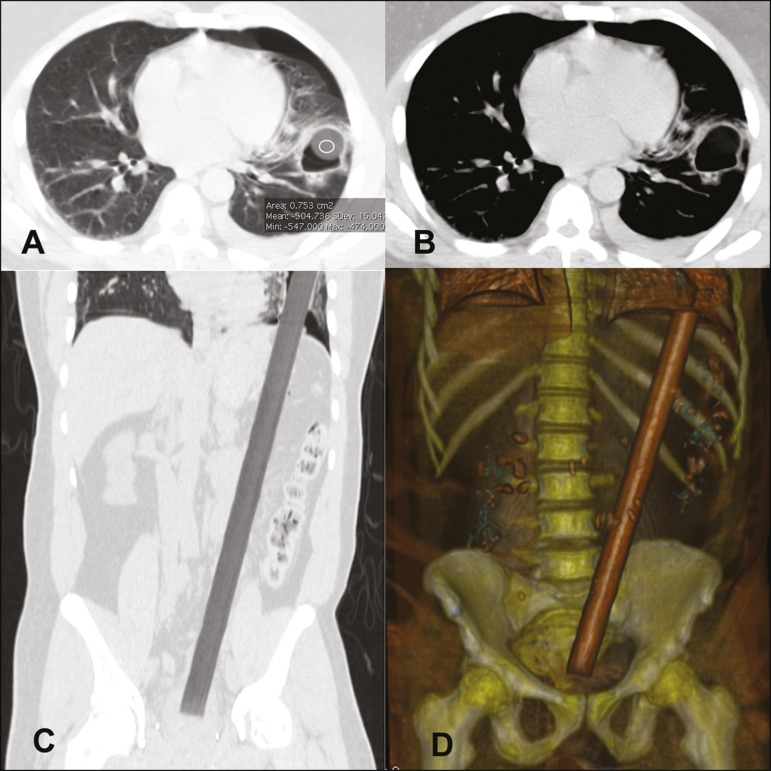
Axial CT with mediastinal and lung window settings (A and B, respectively) showing a laceration on the left lower lobe, accompanied by hydropneumothorax. Note also a hypodense (−501 HU) rounded structure within the cavity (in B). Coronal reconstruction (C) and volume-rendered frontal view (D) demonstrating the extent of the impalement, showing the proximal end of the broomstick in the pelvis and the distal end in the left hemithorax.

Dear Editor,

A 58-year-old male was the victim of physical aggression with impalement by a broomstick. He presented mental confusion, poor response to verbal requests, tachypnea, sweating, and chest pain. Physical examination demonstrated anal bleeding. Thoracoabdominal computed tomography (CT) showed a well-delineated, cylindrical, hypodense (−501 HU) structure extending from the pelvis to the left hemithorax, with pulmonary laceration accompanied by hydropneumothorax (Figure 1). The foreign body measured 40 cm in length and 2.5 cm in width. Emergency midline laparotomy and left thoracotomy revealed the foreign body (a broomstick), extraperitoneal perforation of the rectum, and laceration of the left hemidiaphragm with hemopneumothorax. No significant vascular injury was observed. Colostomy, suture of the diaphragmatic injury, and thoracic drainage were performed. The patient was discharged in good condition after 15 days.

Acute abdominal diseases have been the subject of several recent publications in the radiology literature of Brazil^(1-6)^. Many transanal impalement injuries result from the insertion of foreign bodies (solid elongated objects) into the rectum. Transanal injuries include iatrogenic cases and those related to sexual activities and violent actions. They often involve damage to multiple organs, depending mostly on the direction and nature of the penetrating object^(7-9)^. Impalement injuries are often associated with vascular and visceral damage, entailing significant morbidity and mortality. However, some cases involve no major injuries, potentially because the rounded ends of foreign objects act as blunt tunnelers, resulting in displacement, rather than penetration, of the major visceral and vascular structures^(8-11)^. The clinical diagnosis of these injuries can be challenging. In the case reported here, the patient presented mental confusion and the impaling object was not visible on physical examination. The use of CT allowed us to establish the diagnosis and to predict the extent of the various injuries preoperatively.

In conclusion, combined thoracic and abdominal trauma after rectal impalement is a serious medical situation that calls for the involvement of a multidisciplinary surgical team, with the participation of a thoracic surgeon and an abdominal surgeon. CT is a useful tool for the assessment of retained wooden foreign bodies and for the evaluation of the extent of the injuries.
